# Factors determining the survival of nasopharyngeal carcinoma with lung metastasis alone: does combined modality treatment benefit?

**DOI:** 10.1186/1471-2407-11-370

**Published:** 2011-08-24

**Authors:** Xun Cao, Li-Ru He, Fang-Yun Xie, You-Fang Chen, Zhe-Sheng Wen

**Affiliations:** 1 State Key Laboratory of Oncology in South China, Cancer Center, Sun Yat-Sen University, Guangzhou, China; 2 Department of Thoracic Oncology, Cancer Center, Sun Yat-Sen University, No.651, Dongfeng Road East, Guangzhou, China; 3 Department of Radiation Oncology, Cancer Center, Sun Yat-Sen University, No.651, Dongfeng Road East, Guangzhou, China

## Abstract

**Background:**

Nasopharyngeal carcinoma (NPC) with lung metastasis alone has been reported as a relatively favorable prognostic group, and combined modality treatment might be indicated for selected cases. However, the prognostic factors determining survival of this group and the indication of combined therapy have not been thoroughly studied.

**Methods:**

We retrospectively reviewed 246 patients of NPC with lung metastasis(es) alone presented at diagnosis or as the first failure after primary treatment from 1993 to 2008 in an academic tertiary hospital. Univariate and multivariate survival analyses of post-metastasis survival (PMS) and overall survival (OS) were carried out to determine the prognostic factors.

**Results:**

The 3-year, 5-year, and 10-year of PMS and OS for the whole cohort were 34.3%, 17.0%, 8.6% and 67.8%, 45.4%, 18.5%, respectively. The median PMS (45.6 months *vs*. 23.7 months) and OS (73.7 months *vs*. 46.2 months) of patients treated with combined therapy was significantly longer than that of those treated with chemotherapy alone (*P *< 0.001). Age, disease-free interval (DFI) and treatment modality were evaluated as independent prognostic factors of OS, while only age and treatment modality retain their independent significance in PMS analysis. In stratified survival analysis, compared to chemotherapy alone, combined therapy could benefit the patients with DFI > 1 year, but not those with DFI ≤ 1 year.

**Conclusions:**

Age ≤ 45 years, DFI > 1 year, and the combined therapy were good prognostic factors for NPC patients with lung metastasis(es) alone. The combination of local therapy and the basic chemotherapy should be considered for these patients with DFI > 1 year.

## Background

Nasopharyngeal carcinoma (NPC) is most prevalent in southeastern Asia [1,2], the histology of which is almost World Health Organization (WHO) types III (undifferentiated) [3]. In contrast to other squamous cell carcinoma of the head and neck, NPC is characterized by a high tendency for metastatic dissemination [4,5]. About 5-8% of NPC patients are present with distant metastasis(es) at diagnosis, and 30 to 60% of locally advanced patients will develop distant metastasis(es) and die of disseminated disease [2,4,6]. NPC with distant metastasis makes a very heterogeneous group, of which the post-metastatic survival can vary considerably ranging from weeks to years [7-9].

The lung is a one of the most common organ to which distant metastasis can occur in NPC patients [10,11]. Hui et al reported that, compared with other metastatic patterns, those with lung metastasis(es) alone presented a significantly better overall survival [7]. Others have reported up to 60 months disease-free survival in patients with solitary intrathoracic metastasis treated aggressively with combined therapy [8,12,13]. Thus, we postulate that it may be associated with a unique biologic behavior in NPC patients with lung metastasis(es) alone. However, to our best knowledge, the mechanism behind the phenomenon and the precise predictive factors for intervention and prognosis of this group haven't been clearly identified.

To date, the role of chemotherapy is well established for metastatic NPC, but the objective response is still far from satisfied [14-19]. Although several publications have shown in small cohorts that NPC patients with lung metastasis(es) alone may benefit from combined therapy, no data exists in larger patient cohorts [8,20-22]. We set out this retrospective study thought to add more evidences to help define the predictive factors of this group in a large cohort, and to facilitate the selection of the appropriate group to receive combined modality treatment for a better survival.

## Methods

### Patients

A database was prospectively established for the purpose of this analysis. Between 1993 and 2008, a total of 246 NPC patients with lung metastasis(es) (either present at diagnosis or at the first failure after receiving primary treatment) in Sun Yat-Sen University Cancer Center were consecutively enrolled. All the patients were pathologically confirmed NPC, and evaluated as complete remission for local-regional disease after primary treatment of radiotherapy with or without chemotherapy. Lung metastasis(es) was routinely determined by chest X-ray and/or computed tomography (CT). When both X-ray and/or CT were insufficient to confirm lung metastasis(es), the pathological confirmation by biopsy was then carried out. Patients who have preexisting malignant disease or a second primary tumor, or those with extra-pulmonary metastases were excluded. The study was approved by the medical ethics committee of Sun Yat-Sen University Cancer Center.

The stages of the disease were classified according to the Tumor-Node-Metastasis system (NCCN 2007). The differentiation status and histotype of the disease were classified according to the World Health Organization (WHO) classification for NPC. The characteristics of the 246 NPC patients enrolled in the study are shown in Table 1.

**Table 1:** Characteristics of 246 NPC patients with lung metastasis(es) alone at diagnosis or the first failure.

Characteristics	No. of patients (%)
Age (years) *^a^*	
≤ 45	130 (52.8)
> 45	116 (47.2)
Gender	
Male	196 (79.7)
Female	50 (20.3)
Histology	
WHO type I	3 (1.2)
WHO type II	7 (2.8)
WHO type III	236 (96.0)
EBV VCA-lgA*^b^*	
≤ 320:1*^c^*	100 (40.7)
> 320:1	70 (28.5)
EBV EA-lgA*^b^*	
≤ 40:1*^d^*	112 (45.5)
> 40:1	58 (23.6)
UICC T classification	
T1-2	97 (39.4)
T3-4	149 (60.6)
UICC N classification	
N0	41 (16.7)
N1-3	205 (83.3)
UICC M classification	
M0	201 (81.7)
M1	45 (18.3)
Lung metastasi*s*	
DFI (years)	
≤ 1	92 (37.4)
1-3	86 (35.0)
> 3	68 (27.6)
Metastasis site	
Unilateral	118 (48.0)
Bilateral	128 (52.0)
Metastasis number	
Solitary	82 (33.3)
Multiple	164 (66.7)
Metastasis size (cm) *^e^*	
≤ 2	147 (59.8)
> 2	99 (40.2)

Abbreviation: NPC, nasopharyngeal carcinoma; WHO, World Health Organization; UICC, International Union Against Cancer; DFI, disease-free interval.

*^a ^*Median age;

*^b ^*Data was available for 170 cases;

*^c ^*Median EBV VCA lgA;

*^d ^*Median EBV EA lgA;

^*e *^Median metastasis size.

### Treatments

For patients presented with lung metastasis(es) at diagnosis (M1), 6 cycles of chemotherapy with Cisplatin/5-fluorouracil (PF) regimen were administered before concurrent chemoradiotherapy. The PF chemotherapy regimen consisted of 20 to 30 mg/m^2 ^cisplatin intravenous bolus on days 1 to 3 and 800 to 1,000 mg/m^2^/24 hours continuous intravenous infusion of 5-fluorouracil on days 1 to 4 of each cycle, repeated every 21 days. For those with M0 stage, radical radiotherapy or concurrent chemoradiotherapy with the Cisplatin-based regimen were performed for early stage or locally advanced NPC patients, respectively. During the treatments, new metastatic lesions were found in none of the patients, and all of them received external beam radiotherapy by a 6 MV linear accelerator. All patients had planning computerized tomography of the head and neck performed with patient in the treatment position. Computerized tomography-assisted radiation treatment planning was obtained before the initiation of radiotherapy. A dose of 68-70 Gy/6.5-7 weeks was normally given to the primary tumor. A does of 64-66 Gy/6-7 weeks to the involved neck nodes, whereas the does for node-negative neck was 50 Gy/5-5.5 weeks. Therapeutic efficacy was assessed 3-6 months after the primary treatment, according to the Response Evaluation Criteria in Solid Tumors (RECIST) criteria.

The patients were followed up in the out-patient clinics, where diagnostic examinations consisting of nasopharyngeal and neck magnetic resonance imaging (MRI), chest x-ray and/or CT, abdominal ultrasonography and bone scan were performed every 3-6 months for the first 3 years and finally annually thereafter to detect local recurrence and/or metastasis. For those present with lung metastasis(es) alone as the first failure, 4-6 cycles of palliative chemotherapy with DDP based regimen was administered. Local therapies such as surgery or radiotherapy served as options for those still with metastatic lesions limited in lung after chemotherapy.

### Statistical analysis

Statistical analysis was performed using SPSS 13.0 package (SPSS Standard version 13.0, SPSS Inc, Chicago, IL). Chi-square test was used to compare the difference of categorical variables. Disease-free interval (DFI) was calculated from the completion of initial treatment to the time when lung metastasis(es) was identified. Overall survival (OS) or post-metastasis survival (PMS) was defined as from the date of completing the initial treatment or the date of lung metastasis(es) identified to the date of death or the last follow-up, respectively. The survival curves were calculated by the Kaplan-Meier method and compared using the log-rank test. Multivariate survival analysis was performed on all parameters that were found significant on univariate analysis using the Cox regression model. *P *values < 0.05 were considered significant.

## Results

### Patients' characteristics and treatments

The clinical and pathological characteristics of the 246 NPC patients with lung metastasis(es) alone in this study are listed in Table 1. Among 246 patients, 45 patients were diagnosed with NPC along with lung metastasis(es) while the other 201 cases developed lung metastasis(es) after primary treatment. Single radiotherapy, concurrent chemoradiotherapy and 6 cycles of chemotherapy followed by concurrent chemoradiotherapy were performed in 55, 146 and 45 cases, respectively. Each of the patients had a complete remission (CR) for nasopharynx and the involved cervical nodes after the primary treatment.

After the lung metastasis(es), all the patients received at least 4-6 cycles of chemotherapy, 126 cases of which received ≤ 6 cycles, the other 120 cases received > 6 cycles. CR for the metastatic lung lesions evaluated by CT was obtained in 4.9% (12/246) of the patients after chemotherapy; for those CR was not obtained, palliative metastasectomy or radiotherapy was served as an option, and was performed in 27 and 37 of the 234 patients. Local-regional recurrence along with or after lung metastasis(es) was observed in 31 patients out of the total 246 cases. The 23 cases with local nasopharyngeal recurrence had palliative radiotherapy and the other 8 cases with regional lymph nodes recurrence received regional neck lymph nodes dissection.

The treatment of lung metastasis(es) and/or local recurrence were tolerated. The Common Terminology Criteria for Adverse Events (CTCAE) was used to evaluate the acute toxicity. There were no treatment-related deaths and no grade 4 toxicity. The acute toxicities were listed in Table 2. Twelve patients experienced long-term toxicities such as xerostomia (six patients), mucositis (three patients), radiation pneumonitis (two patients) and sensorineural hearing loss (one patient).

**Table 2:** Grade 3/4 acute toxicity according to CTCAE.

Acute toxicity	No. of patients	%
Evaluable patients	198	
Leukopenia		
Grade 3	65	32.8
Grade 4	0	0
Thrombocytopenia		
Grade 3	19	9.6
Grade 4	0	0
Anemia		
Grade 3	30	15.2
Grade 4	0	0
Vomitting		
Grade 3	22	11.1
Grade 4	0	0
Mucositis		
Grade 3	25	12.6
Grade 4	0	0
Diarrhea		
Grade 3	6	3.0
Grade 4	0	0
Stomatitis		
Grade 3	6	3.0
Grade 4	0	0
Renal toxicity		
Grade 3	0	0
Grade 4	0	0

Abbreviation: CTCAE, Common Terminology Criteria for Adverse Events.

### Association between clinical characteristics and DFI

The mean and median DFI for the primary M0 cases were 34.4 months and 22.7 months, respectively, ranging from 2.3 months to 184.9 months. The mean and median DFI for the primary M1 cases were calculated as 0. In total, there are 92, 86, and 68 cases with a DFI less than 1 year, 1-3 years and greater than 3 years, respectively. As shown in Table 3, patients with age less than 45 years, T1-2 and N0 classification seems to have a longer DFI before lung metastasis(es) developed than those with age greater than 45 years, T3-4 and N1-3 classification.

**Table 3:** Association between clinical characteristics and DFI in 246 NPC patients with lung metastasis(es) alone at diagnosis or the first failure.

	Disease-free interval			
	
Characteristics	≤ 1 year	1-3 years	≥ 3 years	***P ***^*a*^
Age (years)				0.018
≤ 45	39 (30.0)	47 (36.2)	44 (33.8)	
> 45	53 (45.7)	39 (33.6)	24 (20.7)	
Gender				0.701
Male	75 (38.3)	66 (33.6)	55 (28.1)	
Female	17 (34.0)	20 (40.0)	13 (26.0)	
EBV VCA-lgA*^b^*				0.624
≤ 320:1*^c^*	35 (35.0)	37 (37.0)	28 (28.0)	
>320:1	30 (42.9)	22 (31.4)	18 (25.7)	
EBV EA-lgA*^b^*				0.826
≤ 40:1*^d^*	42 (37.5)	38 (33.9)	32 (28.6)	
> 40:1	23 (39.7)	21 (36.2)	14 (24.1)	
UICC T classification				0.001
T1-2	25 (25.8)	33 (34.0)	39 (40.2)	
T3-4	67 (45.0)	53 (35.5)	29 (19.5)	
UICC N classification				0.012
N0	10 (24.4)	12 (29.3)	19 (46.3)	
N1-3	82 (40.0)	74 (36.1)	49 (23.9)	

Abbreviation: NPC, nasopharyngeal carcinoma; DFI, disease-free interval; UICC, International Union Against Cancer.

^*a *^Chi-square test;

*^b ^*Data was available for 170 cases;

^*c *^Median EBV VCA lgA;

*^d ^*Median EBV EA lgA.

### Survival status

**Figure 1:**
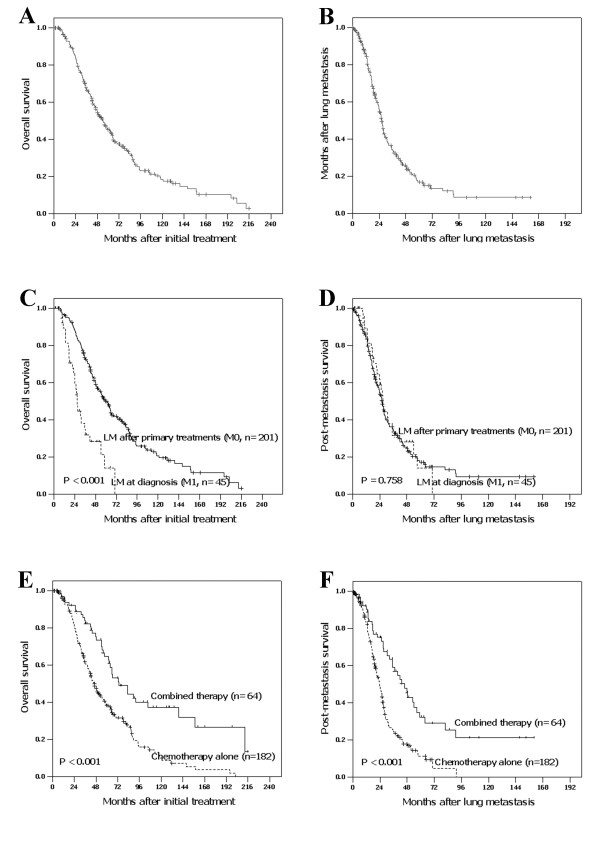
**Survival analysis according to different groups**. Overall survival (OS) and post-metastasis survival (PMS) curves for the whole cohort (A and B) and stratified by M classification (C and D) and treatment modalities (E and F).

The median observation period was 45.8 months (range, 3.2-218.7 months) for the whole cohort, and 170 deaths were observed. The 3-year, 5-year and 10-year OS and PMS for the entire cohort of patients were 67.8%, 45.4%, 18.5% and 34.3%, 17.0%, 8.6%, respectively (Figure 1A and 1B). The median survival time of patients with M0 classification compared with M1 classification was significantly longer in OS (61.7 months *vs*. 26.8 months, *P *< 0.001) but similar in PMS (26.2 months *vs*. 26.8 months, *P *= 0.758, Figure 1C and 1D). The median OS and PMS for patients treated with combined therapy and chemotherapy alone were 73.7 months, 46.2 months (*P *< 0.001) and 45.6 months, 23.7 months (*P *< 0.001), respectively (Figure 1E and 1F).

### Prognostic factors of survival

In univariate analysis, age, EBV VCA-lgA, DFI, metastasis site, metastasis number, local-regional recurrence, treatment modality and chemotherapy effect were evaluated as predicting factors for PMS; while only age and treatment modality retained their significance in multivariate analysis (Table 4). Similarly, age, T classification, N classification, M classification, DFI, metastasis site, metastasis number, metastasis size and treatment modality were evaluated as prognostic factors for OS; while only age, DFI and treatment modality retained their significance in multivariate analysis (Table 4).

**Table 4:** Prognostic variables of 246 NPC patients with lung metastasis(es) alone at diagnosis or the first failure.

	Post-metastasis survival				Overall survivall			
	
Variables	Univariate Analysis	***P***^*a*^	Multivariate Analysis	***P ***^*a*^	Univariate Analysis	***P ***^*a*^	Multivariate Analysis	***P***^*a*^
Age	1.485 (1.097-2.010)	0.010	1.537 (1.038-2.277)	0.032	1.734 (1.277-2.355)	< 0.001	1.424 (1.027-1.974)	0.034
Sex	1.018 (0.712-1.458)	0.921	-	-	1.022 (0.714-1.463)	0.905	-	-
T classification	1.298 (0.948-1.778)	0.104	-	-	1.774 (1.281-2.456)	0.001	1.326 (0.936-1.876)	0.112
N classification	1.224 (0.809-1.852)	0.339	-	-	1.758 (1.150-2.688)	0.009	1.395 (0.905-2.150)	0.131
M classification	0.938 (0.625-1.408)	0.758	-	-	3.010 (1.965-4.612)	< 0.001	1.290 (0.766-2.172)	0.338
EBV VCA-lgA	1.490 (1.033-2.148)	0.033	1.342 (0.920-1.958)	0.127	1.348 (0.935-1.942)	0.110	-	-
EBV EA-lgA	1.171 (0.803-1.710)	0.412	-	-	1.199 (0.820-1.753)	0.349	-	-
DFI	0.818 (0.676-0.991)	0.040	1.019 (0.796-1.304)	0.881	0.326 (0.263-0.404)	< 0.001	0.379 (0.294-0.490)	< 0.001
Metastasis site	1.846 (1.357-2.512)	< 0.001	1.295 (0.779-2.153)	0.319	1.964 (1.439-2.682)	< 0.001	1.561 (0.967-2.520)	0.068
Metastasis number	1.813 (1.295-2.539)	0.001	1.274 (0.705-2.301)	0.422	1.970 (1.403-2.768)	< 0.001	1.284 (0.761-2.097)	0.365
Metastasis size	0.952 (0.699-1.296)	0.754	-	-	0.689 (0.505-0.941)	0.019	0.922 (0.664-1.281)	0.629
Recurrence	1.785 (1.125-2.832)	0.014	1.429 (0.778-2.625)	0.250	1.197 (0.755-1.896)	0.444	-	-
Treatment modality^b^	0.440 (0.302-0.641)	< 0.001	0.583 (0.355-0.960)	0.034	0.454 (0.321-0.660)	< 0.001	0.566 (0.374-0.857)	0.007
Chemotherapy cycle^c^	0.828 (0.612-1.122)	0.828	-	-	1.143 (0.842-1.552)	0.392	-	-
-Chemotherapy effect^d^	2.305 (1.016-5.228)	0.046	1.243 (0.511-3.026)	0.632	1.603 (0.708-3.627)	0.258	-	-

Abbreviation: NPC, nasopharyngeal carcinoma; DFI, disease-free interval.

*^a ^*Cox proportional hazards analysis.

*^b ^*Chemotherapy alone *vs*. combined therapy;

*^c ^*chemotherapy cycle < 6 *vs*. chemotherapy cycle ≥ 6;

*^d ^*Complete remission *vs*. non-complete remission.

**Figure 2:**
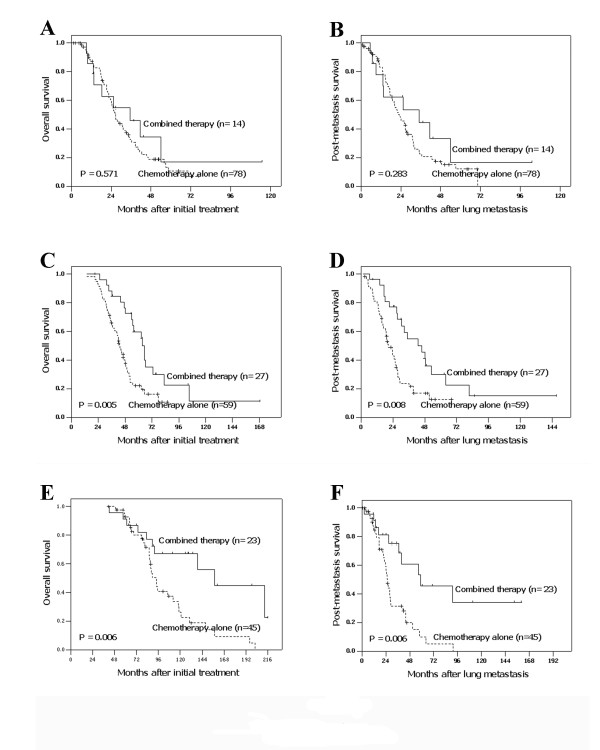
**Stratified analysis according to age, DFI and treatment modality**. Overall survival (OS) and post-metastasis survival (PMS) curves stratified by treatment modalities in different disease-free intervals (DFI): DFI < 1 year (A and B), DFI = 1-3 years (C and D) and DFI > 3 years (E and F).

Since age and DFI together with treatment modality were found to be important prognostic factors for patient survival, we next performed stratified analysis according to age and DFI to find out which subgroups of NPC patients with lung metastasis(es) alone will benefit from the combined therapy modality. The results showed that patients treated with combined therapy presented a better survival than those treated only with chemotherapy in both age less than 45 years and age greater than 45 years subgroups (Table 5). Treatment modality could also stratify the outcome of patients with DFI greater than 1 year, but not those with DFI less than 1 year (Table 5, Figure 2).

**Table 5:** Stratified analysis in 246 NPC patients with lung metastasis(es) alone at diagnosis or the first failure.

		PMS		OS	
		
Variables	Cases	HR (95% CI)	***P ***^*a*^	HR (95% CI)	***P***^*a*^
Age ≤ 45 years			0.004		0.005
Chemo alone	90	1		1	
Chemo+local	40	0.484 (0.293-0.798)		0.485 (0.292-0.805)	
Age > 45 years			0.005		0.006
Chemo alone	92	1		1	
Chemo+local	24	0.434 (0.242-0.779)		0.451 (0.257-0.792)	
DFI < 1 year			0.283		0.571
Chemo alone	78	1		1	
Chemo+local	14	0.676 (0.332-1.380)		0.814 (0.400-1.656)	
DFI = 1~3 years			0.008		0.005
Chemo alone	59	1		1	
Chemo+local	27	0.460 (0.260-0.815)		0.429 (0.239-0.770)	
DFI > 3 years			0.006		0.006
Chemo alone	45	1		1	
Chemo+local	23	0.353 (0.168-0.739)		0.342 (0.160-0.730)	

Abbreviation: NPC, nasopharyngeal carcinoma; PMS, post-metastasis survival; OS, overall survival; HR, hazard ratio; 95% CI, 95% confidence interval; DFI, disease-free interval.

*^a ^*Cox proportional hazards analysis.

## Discussion

Although the prognosis of metastatic NPC is still quite poor, it has been well accepted that the survival of metastatic NPC can be highly variable and long-term survival is possible in some patients [7-9,17,23,24]. Particularly, NPC patients with lung metastasis(es) alone were reported as a distinctive group with a good prognosis compared with other type of metastasis [7,8]. Thus, the identification of prognostic factors of this group will be of great importance from both a therapeutic and research point of view. In this study, we presented the long-term outcome and prognostic indicators of survival in a large cohort of NPC patients with lung metastasis(es) alone.

The 3-year OS and PMS reported in metastatic NPC patients with different series were less than 40% and 20%, respectively [9]. In the present study, we reported a much better prognosis in 246 NPC patients with lung metastasis(es) alone, with a 3-year OS of 67.8% and a 3-year PMS of 34.3%. Our results concurred closely with another study of lung metastasis of NPC in Hong Kong that NPC patients with lung metastasis(es) alone (*n *= 41) appeared to have a distinctively better prognosis, with a hazards ratio (HR) of 0.41 compared with other types of metastasis(es) [7]. In other studies, intrathoracic metastasis(es) (lung and/or mediastinal nodes) was considered as a good prognostic factor in metastatic NPC [8,20,21]. However, to our knowledge, no literature has identified the precise predictive factors for the prognosis in this specific group.

It was demonstrated in our study that young age (≤ 45 years), long DFI (> 1 year), unilateral metastasis, solitary metastasis and combined therapy were good prognostic factors in terms of both OS and PMS based on univariate survival analysis, while only young age, long DFI and combined therapy were found to be independent good prognostic factors of OS according to multivariate analysis. These factors have also been indicated in other studies as good prognosis in patients with lung metastasis(es) from head and neck cancers [4,11,25,26]. For young age, we cannot explain clearly why it is a good prognostic factor, but it may be related to the significant association between young age and long DFI found in the present study. In another hand, young patients usually have better performance status to tolerate the side effect of aggressive combined therapy, and react positively towards the treatment than old patients, and thus they are more likely to accept the treatment after the discovery of lung metastasis(es) than elderly patients.

DFI has long been identified as an important prognostic factor for patient outcome, but the significant cutoff points of DFI are still uncertain according to the literatures [4,8,11,27-29]. In NPC, Teo et al reported that the OS of patients presenting with distant metastasis(es) abinitio (M1, DFI = 0) is much worse than those developing distant metastasis(es) after primary radiotherapy (M0, DFI > 0) [8]. In our study, DFI = 0 (M1) was also found to a poor prognostic factor of OS of NPC patients in univariate analysis, but there was no difference of PMS between DFI = 0 months (M1) and DFI > 0 months (M0) groups. Another reported cutoff of DFI < 6 months, which was identified by Ong et al as an independent prognostic factor of PMS in metastatic NPC; while in another study conducted by Khanfir et al, no difference of PMS between groups with DFI ≤ 6 months and DFI > 6 months was observed [4,11]. It's believed that different types of metastatic NPC might correspond with different significant cutoff points of DFI, which might partly contribute the variances of DFI cutoff points reported in the literatures. In our study, we proposed that DFI ≤ 1 year, 1-3 years and > 3 years were the proper cutoff values to distinguish both the OS and PMS in NPC patients with lung metastasis(es) alone. Patients with young age (≤ 45 years), early T classification, and without lymph node metastasis would be likely to have longer DFI.

Treatment modality is another important independent prognostic factor in our study. We demonstrated that, compared to single chemotherapy, combined therapy improved both the OS and PMS for NPC patients with lung metastasis(es) alone. Geara et al reported similar results in 103 metastatic NPC patients who treated with radiotherapy and chemotherapy (2 yeras-OS, 45%) for their metastases had a significantly better survival than those received no treatment (2 years-OS, 14%) or single chemotherapy (2 years-OS, 18%, *P *= 0.001) [22]. In addition, some long-term survivors aggressively treated with pulmonary metastasectomy in combination with chemotherapy were also reported in NPC patients with intrathoracic metastasis(es) alone [10,12,13,20,28-30]. Collectively, these data suggests an indication for combined therapy in selected metastatic NPC patients. Thus, it seems necessary to further evaluate which subgroup of patients would benefit from it. In our stratified survival analysis, treatment modality was found to have improved the outcome of patients with DFI greater than 1 year, but not those with DFI less than 1 year.

There are several limitations to our study. Our study is a retrospective study, relied exclusively on a single-institutional database. A larger scale, prospective, multi-center study is needed to confirm our results.

## Conclusions

In conclusion, our study is the first retrospective study in southern China to identify the prognostic indicators of long-term survival in a large group of NPC patients with lung metastasis(es) alone. Based on our results, age less than 45 years, DFI greater than 1 year and the combined modality treatment were good prognostic factors. The combined therapy should be highly considered for lung metastatic NPC patient with DFI greater than 1 year to achieve better survival.

## Competing interests

The authors declare that they have no competing interests.

## Authors' contributions

XC performed the statistical analysis, drafted the manuscript and participated in the sequence alignment. LRH participated in the design of the study and participated in the sequence alignment. FYX participated in the sequence alignment. YFC carried out data acquisition. ZSW conceived of the study, and participated in its design and coordination and helped to draft the manuscript. All authors read and approved the final manuscript.

## Pre-publication history

The pre-publication history for this paper can be accessed here:

http://www.biomedcentral.com/1471-2407/11/370/prepub
